# Mother: a maternal online technology for health care dataset

**DOI:** 10.1186/s13104-025-07230-2

**Published:** 2025-04-08

**Authors:** Odongo Steven Eyobu, Brian Angoda Nyanga, Lukman Bukenya, Daniel Ongom, Tonny J. Oyana

**Affiliations:** 1 Geospatial Data and Computational Intelligence lab, College of Computing and Information Science, Makerere University, P.O Box, 7062, Kampala, Uganda; 2 Department of Networks, School of Computing and Information Technology, Makerere University, P.O Box, 7062, Kampala, Uganda

**Keywords:** Maternal health, Pregnancy, Question and answer knowledge base, Electronic health (E-health), Conversational chatbots

## Abstract

**Objectives:**

These data enable the development of both textual and speech based conversational machine learning models that can be used by expectant mothers to provide answers to challenges they face during the different trimesters of their pregnancy. Such models are key to the improvement of the lives of pregnant mothers, specifically in low resourced settings where doctors advise is limited by access to hospitals and language barrier. These data were used to develop a conversational chatbot model tailored for mothers in their first, second and third trimesters of pregnancy.

**Data description:**

503 question and answer pairs on maternal health were collected through a survey of challenges facing pregnant mothers in a rural and semi-urban area of Uganda. The answers to the questions were provided and validated by professional medical personnel. The participants were purposively sampled, focusing on women in their 1st, 2nd and 3rd trimesters, with a 94% response rate. The dataset addresses common health concerns, symptoms, and conditions associated with pregnancy, particularly for women without immediate access to medical personnel. It targets maternal health outcomes such as pregnancy, morbidity, and mortality, specifically among women of reproductive age.

## Objective

According to a 2019 UNICEF report [1], women in sub-Saharan Africa are fifty times more likely to die from childbirth than women in high-income countries. This is attributed to various factors including limited access to healthcare facilities and professional doctors, lack of emergency care, healthcare information and malnutrition. This article presents a comprehensive question-and-answer dataset [2] for maternal health, designed to enable the development of a conversation chatbot that provides healthcare information to support pregnant mothers with limited access to doctors whenever needed particularly in resource constrained rural areas. The knowledge base [2] provides answers to various challenges of maternal health, including prenatal care, nutrition, pregnancy complications, childbirth, postpartum care, and maternal wellness. With approximately 800 women dying daily due to pregnancy-related complications in rural Africa [3], this dataset [2] serves as a foundation for building intelligent data-driven conversational models to run a chatbot that directly supports pregnant mothers with instant, immediate and accurate on-demand information concerning their healthcare needs. It is envisaged that the resultant conversational chatbots will be able to receive queries and deliver health care information in preferred local languages to take care of the language diversities of rural populations that do not understand English particularly in sub-Saharan Africa. By empowering expectant mothers with reliable information, particularly in low- and middle-income countries, there is a great opportunity to contribute to a reduction in maternal mortality and improving maternal health outcomes.

## Data description

Questions on challenges and lifestyle during pregnancy from 500 expectant mothers were compiled and answers to each question were provided by medical professionals to formulate the question-and-answer pair textual dataset. The age range of the expectant mothers was 20–50 years. The data collected clearly shows pregnancy challenges associated with women in rural settings. These are mainly associated with nutrition challenges, antenatal care, and postpartum care.

Participants were purposively sampled, focusing on women in their 1st, 2nd and 3rd trimesters, with a 94% response rate. 161 mothers were in their first trimester, 142 mothers were in their second trimester, 197 mothers were in their 3rd trimester.

After collecting the data, preprocessing steps were performed to realign the questions and answers into clear English sentences to enhance readability of the texts. Question and answer pairs that were similar were grouped to form patterns and responses respectively. The patterns represented the questions that have the same meaning. The responses represented the answers that had the same meaning. These patterns and responses were further given tags and contexts. The tags were used to show the general topic that the questions and answers addressed.

The contexts on the other hand represented the specific topic that the questions and answers were addressing. Intents were then created by grouping tags, contexts, patterns and responses. This allowed normal question and answer pairs to be modeled into a dataset that can be used to train a BERT [4] model for a chat bot. The reason for having multiple patterns for some questions is because a user interacting with a chat bot may ask a question in different ways and hence the model would have to decipher exactly what the user wants to know and be able to give an appropriate response. Also, in cases where a response could be framed in different ways, multiple responses were created to provide a variety of answers to a given question. Questions that contained single-word answers were rephrased by adding more words to elaborate the answer so that a user could better understand the output from the model.

Data file 1 [2] is: Intents (1) id, (2) tag, (3) context_set where id is the unique identifier for an intent, tag is a general topic about the intent and context_set is a specific topic about the intent.

Data file 2 [2]: Patterns (1) id, (2) intent_id, (3) content, where id is the unique identifier of a pattern, intent_id represents the intent that the pattern belongs to and content is the actual question.

Data file 3 [2]: Responses (1) id, (2) intent_id, (3) content, where id is the unique identifier of a response, intent_id represents the intent that the response belongs to and content is the actual answer.

Data file 4 [2]: Question and answer pairs.

Data file 5 [2]: Question and answer pairs transformed into intents, patterns and responses (Table 1).

**Table 1:** Data file descriptions

Label	Name of data file/data set	File types (file extension)	Data repository and identifier (DOI or accession number)	Reference
Data file 1	intents	Excel (.csv)	Harvard Dataverse 10.7910/DVN/EZLCH3	[2]
Data file 2	patterns	Excel (.csv)	Harvard Dataverse 10.7910/DVN/EZLCH3	[2]
Data file 3	responses	Excel (.csv)	Harvard Dataverse 10.7910/DVN/EZLCH3	[2]
Data file 4	mother_question_and_answer_pairs_data	JSON	Harvard Dataverse 10.7910/DVN/EZLCH3	[2]
Data file 5	mother_intents_patterns_responses_data	JSON	Harvard Dataverse 10.7910/DVN/EZLCH3	[2]

## Limitations

The dataset [2] is presented in English and therefore requires a translator in order for it to be used in English language constrained populations of expectant mothers.

The dataset [2] is limited to responses from only 500 participants, which may not fully capture the diverse challenges faced by pregnant mothers across various regions and demographics. So, the repository will undergo continuous updates bimonthly.

Applying or use of the dataset for conversational chatbots is not a total replacement of doctors but works as an emergency information dissemination tool in resource constrained areas.

## Data Availability

The question answer dataset developed in this study has been deposited in the Havard Dataverse repository accessible at the following link: 10.7910/DVN/EZLCH3.
